# Supplementary material for: Reconciling Mining with the Conservation of Cave Biodiversity: A Quantitative Baseline to Help Establish Conservation Priorities
Source: PLoS One. 2016 Dec 20;11(12):e0168348. doi: 10.1371/journal.pone.0168348 (PMC5173368; doi:10.1371/journal.pone.0168348)
Supplement: S1 Dataset — (ZIP) [file pone.0168348.s002.zip › Taxa/Serra Norte/SN_2013/N4E/Lista N4E-76.pdf]

CAVIDADE N4E-0076

Classe	Ordem	Fam/Outros	Gên/Outros	Espécie	S-U	U-S	Zon.
Arachnida	Acari	Neothyridae	*Diplothyrus*	sp.1		X	P
Arachnida	Acari	Smarididae		sp.15	X		P
Arachnida	Amblypygi	Phrynidae	*Heterophrynus*	*longicornis*	4	2	E/P
Arachnida	Araneae	Mysmenidae	*Microdipoena*	sp.1		X	P
Arachnida	Araneae	Ochyroceratidae	*Speocera*	sp.1		X	P
Arachnida	Araneae	Pholcidae	*Mesabolivar*	sp.1	X	X	E
Arachnida	Araneae	Pholcidae	*Modisimus*	sp.1	X	X	P
Arachnida	Araneae	Scytodidae	*Scytodes*	jovem		1	P
Arachnida	Araneae	Theraphosidae	aff. *Holothele*	sp.1	2	2	P
Arachnida	Araneae	Theridiidae		jovem	X		P
Arachnida	Araneae	Theridiosomatidae	*Plato*	sp.1	X	X	P
Arachnida	Opiliones	Stygnidae	*Protimesius*	*gracilis*		1	E/P
Arachnida	Pseudoscorpiones			jovem		X	P
Arachnida	Schizomida	Hubbardiidae		jovem		X	P
Chilopoda	Geophilomorpha	Schendylidae		sp.1		1	P
Chilopoda	Scolopendromorpha	Scolopocryptopidae	*Tidops*	jovem		1	P
Diplopoda	Polydesmida	Chelodesmidae		sp.1		1	P
Diplopoda	Polydesmida	Chelodesmidae		sp.2		1	P
Diplopoda	Polydesmida	Chelodesmidae		sp.3		3	P
Entognatha	Collembola	Paronellidae		sp.1		X	P
Entognatha	Collembola	Paronellidae		sp.4		X	P
Entognatha	Diplura	Campodeidae		sp.1		X	P
Insecta	Blattodea	Blaberidae		jovem		2	P
Insecta	Blattodea	Blattellidae		jovem	2	1	P
Insecta	Blattodea	Blattidae		sp.4	2	1	P
Insecta	Blattodea	Polyphagidae		sp.1		4	P
Insecta	Coleoptera	Scydmaenidae		sp.3		X	P
Insecta	Coleoptera	Staphylinidae		sp.1		X	P
Insecta	Diptera	Culicidae	*Culex*	sp.		X	E
Insecta	Diptera	Phoridae	Metopininae	sp.		X	P
Insecta	Diptera	Psychodidae	Phlebotominae	sp.		X	P
Insecta	Heteroptera	Reduviidae	Emesinae	sp.7		2	P
Insecta	Heteroptera	Reduviidae	Reduviinae	sp.	2	5	P
Insecta	Homoptera	Cixiidae		sp.2	X	X	P
Insecta	Hymenoptera	Formicidae	*Camponotus*	sp.2	X		P
Insecta	Hymenoptera	Formicidae	*Pachycondyla*	sp.2	X	X	P
Insecta	Isoptera	Rhinotermitidae	*Coptotermes*	sp.1		X	P
Insecta	Isoptera	Termitidae	*Atlantitermes*	sp.		X	P
Insecta	Isoptera	Termitidae	*Diversitermes*	sp.		X	P
Insecta	Isoptera	Termitidae	*Nasutitermes*	sp.	X	X	P
Insecta	Lepidoptera	Gelechioidea		sp.	X		P
Insecta	Lepidoptera	Noctuoidea		sp.5		2	E
Insecta	Orthoptera	Phalangopsidae	*Phalangopsis*	sp.	17	51	E/P
Insecta	Psocoptera	Epipsocidae	*Epipsocus*	sp.1		X	P
Insecta	Thysanura	Nicoletiidae		sp.1	X		P
Gastropoda	Pulmonata	Subulinidae		sp.4		X	P
Gastropoda	Pulmonata	Systrophiidae	*Happia*	sp.		X	P
Malacostraca	Isopoda	Dubioniscidae		sp.1		X	P
Malacostraca	Isopoda	Philosciidae		sp.2		X	P

Malacostraca	Isopoda	Scleropactidae		sp.1	X		P
Amphibia	Anura	Strabomantidae	*Pristimantis*	cf. *fenestratus*	9	1	E/P
Mammalia	Chiroptera	Furipteridae	*Furipterus*	*horrens*	2		P
Reptilia	Squamata	Gekkonidae	*Thecadactylus*	sp.	2		E/P
